# Impact of propofol versus sevoflurane anesthesia on molecular subtypes and immune checkpoints of glioma during surgery

**DOI:** 10.1002/hsr2.1366

**Published:** 2023-09-11

**Authors:** Shenghua Cen, Guocai Yang, Hongyan Bao, Ze Yu, Lei Liang

**Affiliations:** ^1^ Department of Anesthesiology, Zhoushan Hospital Wenzhou Medical University Zhoushan Zhejiang China; ^2^ Department of Thoracic Surgery, Zhoushan Hospital Wenzhou Medical University Zhoushan Zhejiang China; ^3^ The Laboratory of Cytobiology & Molecular Biology, Zhoushan Hospital Wenzhou Medical University Zhoushan Zhejiang China

**Keywords:** glioma, immune checkpoint, propofol, SERPINI1, sevoflurane, WGCNA

## Abstract

**Background:**

Sevoflurane and propofol are two popular anesthetics used during glioblastoma (GBM) surgery. This investigation compared the molecular subtypes and immune checkpoints of cancer cells following GBM surgery under sevoflurane and propofol anesthesia.

**Method:**

The expression profile data and clinical information of glioma samples of different grades were downloaded from The Cancer Genome Atlas database. Weighted gene coexpression network analysis was used to identify hub modules and key genes related to glioma grades (G2 and G3). The GEO database (GSE179004) was used to retrieve glioma surgical specimens with different anesthetic gene expression profiles. The differential expression of immune checkpoint genes under various anesthetic settings was examined using the R‐ggplot2.

**Results:**

Compared to sevoflurane, propofol significantly downregulated SERPINI1 and CAMK2A expression. These are also important factors in glioma grading. Simultaneously, SERPINI1 and CAMK2A were also significantly related to the prognosis of GBM and lower‐grade glioma patients and acted as potential tumor suppressors. In addition, propofol increases the expression of the immune checkpoint molecule, PD‐L1.

**Conclusions:**

Our study revealed that sevoflurane can more effectively prevent the development of glioma after surgery than propofol, and SERPINI1 can be used as a new independent prognostic factor for glioma.

## 1 INTRODUCTION

With a high degree of malignancy and a poor 5‐year survival rate, glioblastoma (GBM) is the most prevalent primary craniocerebral malignant tumor.^1^ It develops from the canceration of glial cells in the brain and spinal cord.^2^ Currently, a number of known factors, such as the patient's age, tumor grade, gene mutation, extent of surgery, and use of radiotherapy and chemotherapy, all affect the progression of the disease.^3^, ^4^ The emergence and growth of malignant tumors are closely related in a number of important ways. The effects of anesthesia on the immune system of surgery patients will be felt.^5^, ^6^ The choice of anesthetic technique and medicine may also have an impact on patient prognosis.

Anesthesia is a crucial surgical procedure that can help cancer patients feel less stimulated throughout the process and lessen their stress response.^7^, ^8^ Despite the relatively brief anesthetic exposure time during glioma resection, the medications used and associated hemodynamic alterations may also affect the postoperative prognosis of glioma patients. Cancer prognosis is affected by the anesthetic approach chosen.^9^ Recent investigations have demonstrated that anesthetics can alter the biological processes of tumors and immune cells in addition to providing analgesic sedation.^10^, ^11^, ^12^, ^13^

Some anesthetic medications, including sevoflurane, dexmedetomidine, and propofol, may have some impact on the malignant phenotype of tumor cells.^14^, ^15^, ^16^ These anesthetics are thought to be potential tumor suppressors or facilitators; however, it is still unknown how they will affect glioma patients. Sevoflurane and propofol are frequently used anesthetics in therapeutic settings. The impact of these two anesthetics on immunological response and tumor cell behavior varies.^17^, ^18^ Much attention has been paid to which increases the sensitivity to postoperative recurrence and metastasis. To date, the impact of these two anesthetic techniques on postoperative biological and immune function in glioma patients is still unclear. Therefore, this study aimed to compare the effects on molecular biology and immune system of cancer cells between propofol‐ and sevoflurane‐based anesthesia in patients undergoing glioma surgery, and to offer a theoretical foundation for better clinical anesthesia selection.

## 2 MATERIALS AND METHODS

### 2.1 Data collection

The clinical features and gene expression profile GSE179004 were downloaded from the Gene Expression Omnibus (GEO) collection.^19^ Under aseptic conditions, GBM specimens were obtained from neurosurgery for RNA extraction and hybridization on Affymetrix microarrays. GBM specimens were obtained by neurosurgery under aseptic conditions for RNA extraction and hybridization on Affymetrix microarrays. The patients were divided into TIVA (propofol) and INHA (sevoflurane) groups. The specimens used for gene microarray analysis were matched according to the time of anesthesia and operation, grade, tumor location, and pathological type. Tumor (BGM and lower‐grade glioma [LGG]) RNAseq data and corresponding clinical information were obtained from the Cancer Genome Atlas (TCGA) data set.

### 2.2 DEGs screening

Using the R package Limma, we examined the differences between the tumor and normal groups. Genes with |log2 (fold change)| >1 and a corrected *p*‐value of 0.05 were classified as DEGs. All DEGs were displayed as heat maps using the “pheatmap” R tool.

### 2.3 Weighted gene coexpression network analysis (WGCNA) and identification of hub modules

A coexpression network of DEGs was constructed using the WGCNA package in R (3.6.2). The soft criterion was determined using a scale‐free *R* ^2^ > 0.85. The cluster dendrogram and gene network heat map were later produced.^20^ The eigengene values of the modules were established after the grouping tree had been constructed. Subsequently, the WGCNA analysis was completed using the SangerBox 3.0 online tool.

### 2.4 Identifying clinically related modules and genes

A summary of all modules was provided by module eigengenes, which were approximated as synthetic genes expressing the expression profile of all the genes in a given module. The relationship between MEs and clinical traits was also computed (LGG: Grade2 or Grade3). Then, using the value of Pearson's correlation, the gene‐trait significance value (GS), which showed the relative level between these genes and traits, was calculated to discover which genes were most associated with malignancies.

### 2.5 Functional enrichment analysis

To confirm the underlying function of the potential targets more thoroughly, the data were evaluated using functional enrichment. GO is a tool frequently used to functionally annotate genes. KEGG enrichment analysis is a helpful method for learning gene functions and associated high‐level genomic functional information. The GO function of potential targets was examined using the ClusterProfiler tool in R, which also improved the KEGG pathway analysis.

### 2.6 Gene and pathway correlation analysis

To determine the correlation between the samples and the pathway, we gathered the gene sets present in relevant pathways and utilized the ssGSEA algorithm to calculate the enrichment scores of each sample on each pathway in turn. It was possible to determine the association between the gene and pathway by evaluating the correlation between gene expression and pathway scores.

### 2.7 Nomogram model construction

TCGA data set provided RNA‐seq data and clinical details for gliomas. The “forestplot” program was used to create forest plots to show each variable (*p* value, HR, and 95% confidence interval [CI]) after performing univariate and multivariate Cox regression analysis. The overall recurrence rate for X years was predicted using a column plot created using the “rms” package based on the findings of multivariate Cox proportional risk analysis. The column chart offers a graphical representation of various risk variables, making it possible to compute the prognostic risk for a specific patient using the points assigned to each risk factor.

### 2.8 Immune checkpoint analysis

TCGA data set provides RNA‐seq data and clinical details for gliomas. Immune checkpoint genes were SIGLEC15, TIGIT, CD274, HAVCR2, PDCD1, CTLA4, LAG3, and PDCD1LG2. The expression levels of these eight genes were retrieved to track the expression of genes associated with immunological checkpoints. The R software programs ggplot2 and pheatmap were used to generate the aforementioned findings.

### 2.9 ICB response

TIDE assesses two different tumor immune escape pathways: immunosuppressor‐mediated cytotoxic T cells (CTL) rejection and malfunction of tumor‐infiltrating CTL. RNA‐seq data and clinical information of gliomas were obtained from TCGA data set. The TIDE algorithm has been used to predict potential immunotherapy responses.^21^

### 2.10 Statistical analysis

The R software (version 3.6.1) was used for all TCGA data analyses. The expression of SERPINI1 and CAMK2A, survival, and other clinical variables were analyzed using multivariate Cox regression analysis after prognostic factors were chosen using univariate Cox regression analysis.

2.11

2.11. Bioinformatics analysis methods and websites were presented in the Supporting Information data.

## 3 RESULTS

### 3.1 Data preprocessing and identification of DEGs in GBM and LGG samples

**Figure 1:**
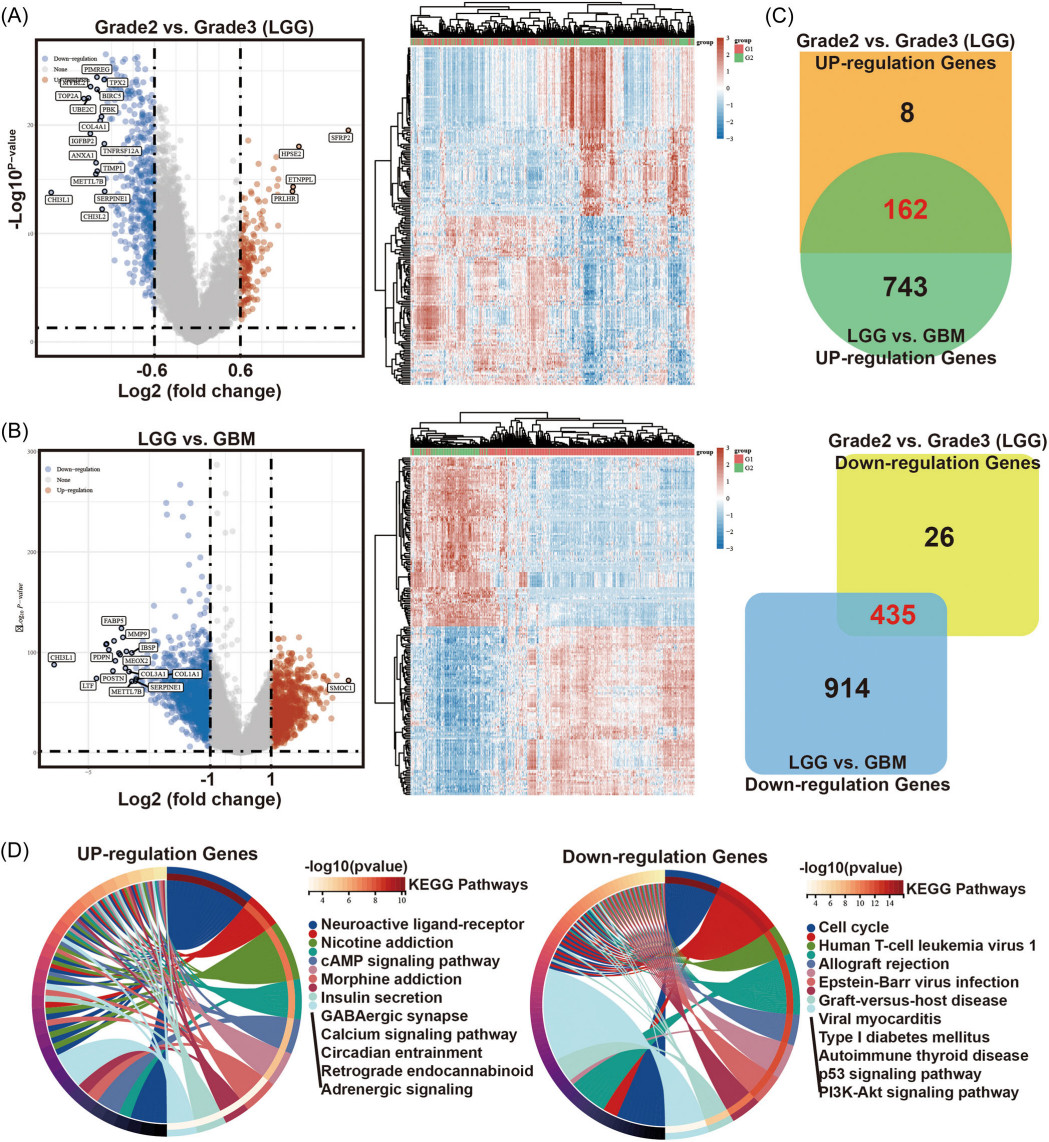
Identification of the DEGs through the classification and grading of glioma. (A) Volcano plots and heat‐maps of the DEGs between Grade2 & Grade3 (LGG), and (B) between LGG & GBM. (C) The Venn diagram of screening DEGs in LGG (Grade2 vs. Grade3) and LGG versus GBM. (D) Significantly enriched the KEGG pathways of the DEGs obtained and visualized through cluster‐Profiler. GBM, glioblastoma; LGG, lower‐grade glioma.

The clinical information and RNA sequencing data of GBM multiforme and brain LGG patients were downloaded from TCGA. RNA sequencing data were obtained from 153 GBM patients and 513 LGG patients. Subsequently, 631 and 2254 DEGs were identified between tumor Grade2 and Grade3 patients with LGG, and between LGG and GBM, respectively (Figure 1A,B and Supporting Information: Figure 1A,B). Glioma Grade3 is more malignant than Grade2, and its prognosis is worse. Similarly, LGG is less malignant than GBM.^22^ Therefore, we intersected the DEGs in two comparison groups (Grade2 vs. Grade3 of LGG, and LGG vs. GBM) and then obtained a list of genes most related to the malignant degree of glioma (162 upregulated and 435 downregulated genes) (Figure 1C).

Functional enrichment analysis showed 20 significantly enriched KEGG pathways and GO terms of the 162 (up) and 435 (down) DEGs, respectively (Figure 1D and Supporting Information: Figure 1C). The results showed that the upregulated genes were mainly involved in insulin secretion and the cAMP signaling pathway. On the other hand, the downregulated genes were closely related to cell growth, such as the cell cycle, p53 signaling pathway, and PI3K‐Akt signaling pathway.

### 3.2 WGCNA analysis and identification of key modules and Hub genes

**Figure 2:**
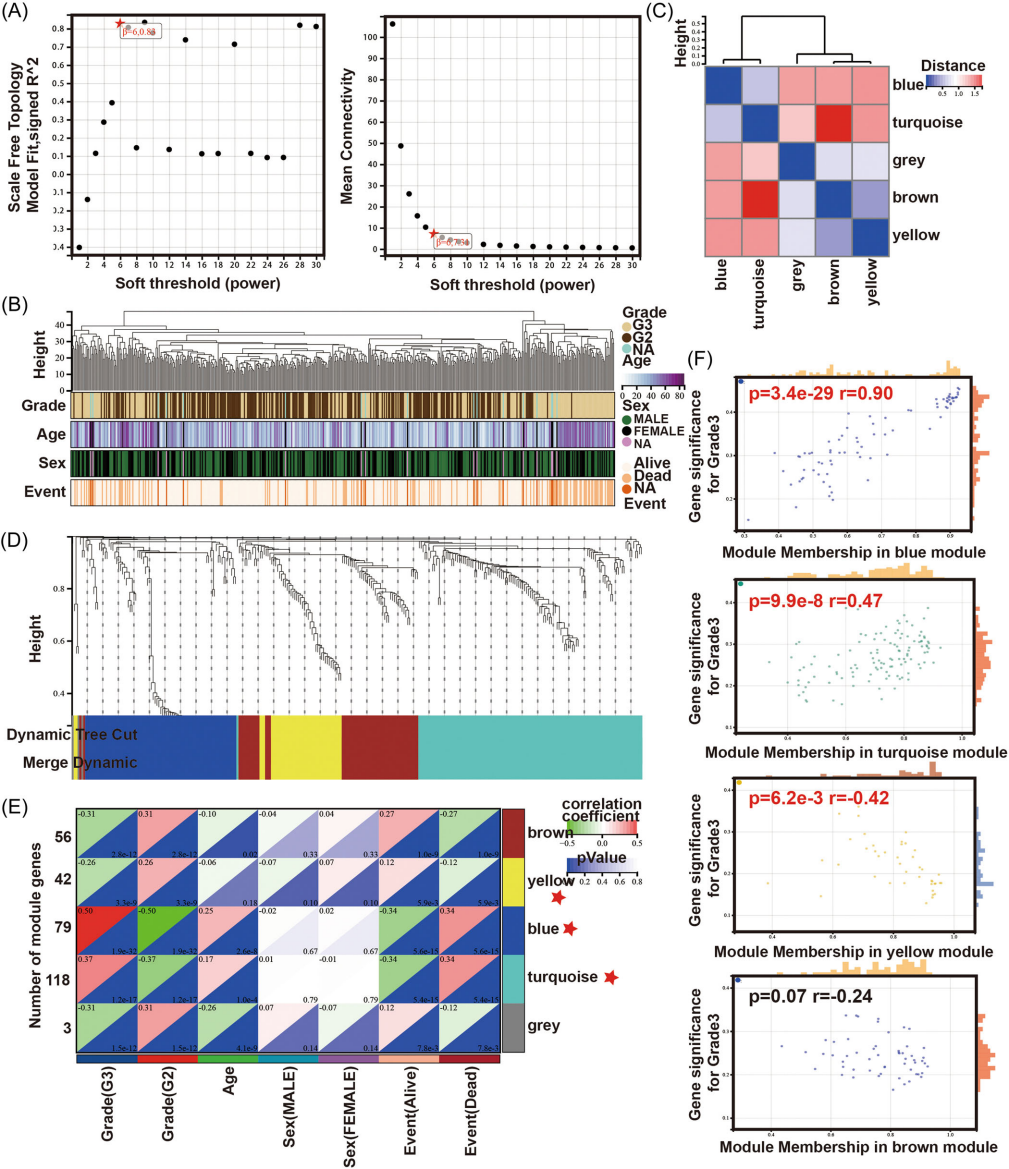
WGCNA of DEGs in LGG. (A) The soft thresholding and correlation coefficient in the scale‐free topology fitting graph. (B) Clustering dendrogram of clinical traits and data from 513 LGG samples. (C) Module eigengene heat‐map of eigengene adjacency. (D) Cluster dendrogram of genes. (E) Heat‐map of correlations between different modules and clinical traits. (F) Scatter plots of GS score and MM. LGG, lower‐grade glioma; WGCNA,weighted gene coexpression network analysis.

Based on 596 DEGs and 516 clinical sample networks from the LGG samples, we constructed a WGCNA coexpression network. As shown in Figure 2A,B, four clinical variables were applied in the WGCNA: tumor grade, age, sex, and events. All DEGs with similar expression patterns were clustered into the same modules, and modules showing a difference in cut height <0.25 were merged. Five coexpression modules were obtained in this procedure, including blue, turquoise, gray, brown, and yellow. Each color module contained different genes (Figure 2C). These modules were independent of each other. The eigengene module values were calculated for each module, and the clustering tree is shown in Figure 2D. The characteristic genes of the turquoise yellow and blue modules were strongly correlated with tumor grade (Figure 2E). In addition, the MM scores were positively correlated with the GS scores in these modules (Figure 2F). These findings indicate that the turquoise and blue modules contribute to tumorigenesis, while the yellow module might delay the progression of glioma. Therefore, these four modules were used to analyze the hub genes.

### 3.3 Identification of the DEGs and pathways via comparing propofol and sevoflurane

**Figure 3:**
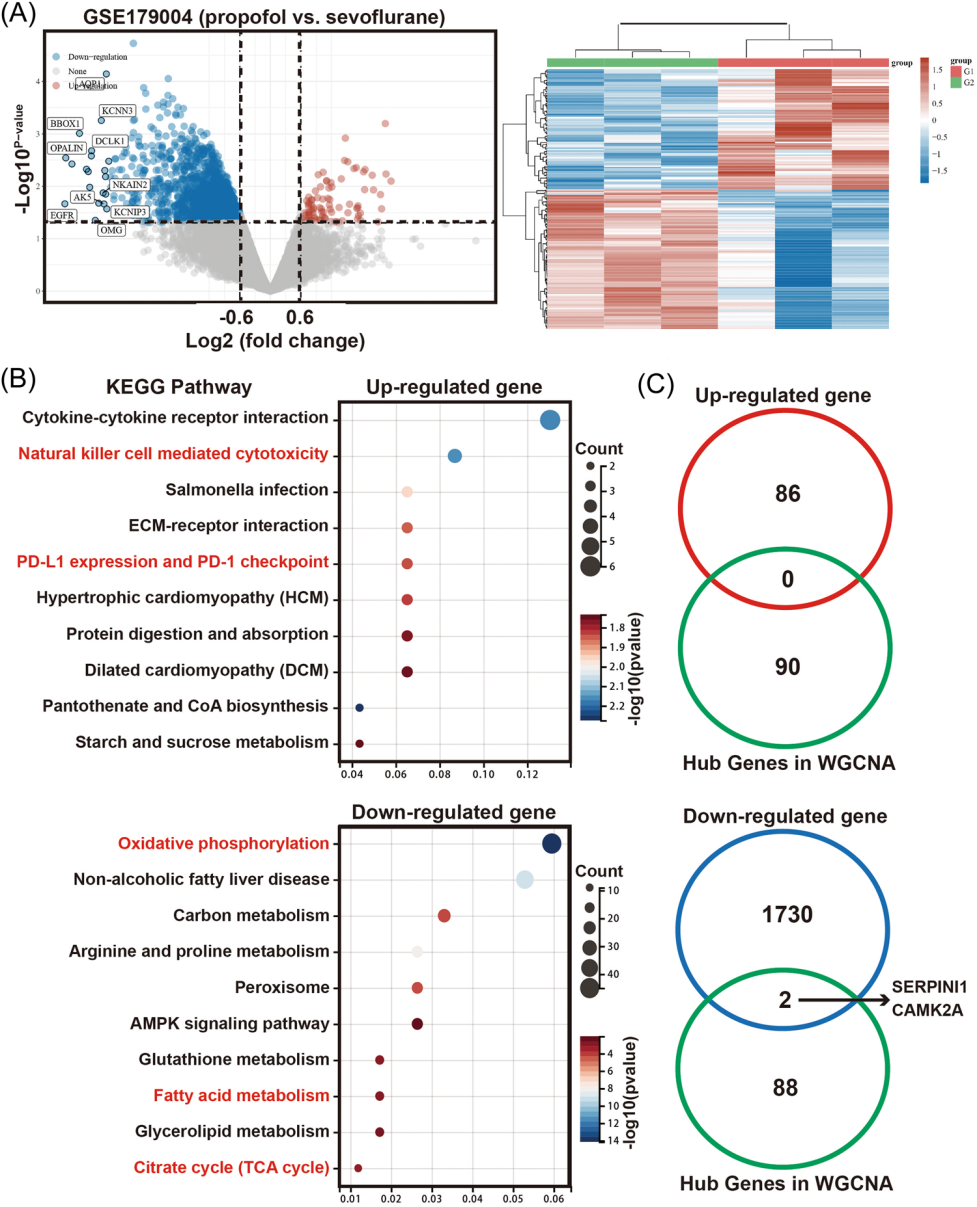
Identification of the DEGs and pathways via comparing propofol and sevoflurane. (A) Volcano plot and heat‐map of the DEGs between propofol and sevoflurane. (B) Significantly enriched the KEGG pathways of the DEGs. (C) Venn diagram of screening genes in DEGs (up‐ and downregulated, respectively) and the Hub genes in WGCNA. WGCNA,weighted gene coexpression network analysis.

To explore the differences in gene expression between propofol and sevoflurane in patients with GBM, we screened the genes that were differentially expressed in the propofol group compared to the sevoflurane group. The data showed that 82 significantly upregulated genes and 1732 downregulated genes were present (Figure 3A).

We then performed a KEGG pathway analysis to understand the differences between propofol and sevoflurane. KEGG pathway analysis showed that the 82 upregulated genes were mainly involved in cytokine‐cytokine receptor interaction, natural killer cell‐mediated cytotoxicity, PD‐L1 expression, and PD‐1 checkpoint. These downregulated genes were mainly enriched in metabolic pathways such as oxidative phosphorylation, glutathione metabolism, TCA cycle, and fatty acid metabolism (Figure 3B). These findings indicate that propofol and sevoflurane have substantial effects on the control of immune checkpoints and cell metabolism. Subsequently, we interacted the screened DEGs with the hub genes in WGCNA and obtained two genes in the downregulated gene set, SERPINI1 and CAMK2A (Figure 3C).

### 3.4 Identification of SERPINI1 and CAMK2A as important genes in glioma

SERPINI1 and CAMK2A were found to be the only two overlapping genes in both groups, indicating that these genes are not only highly correlated with the progression of glioma but also act as important molecular markers during glioma anesthesia.

**Figure 4:**
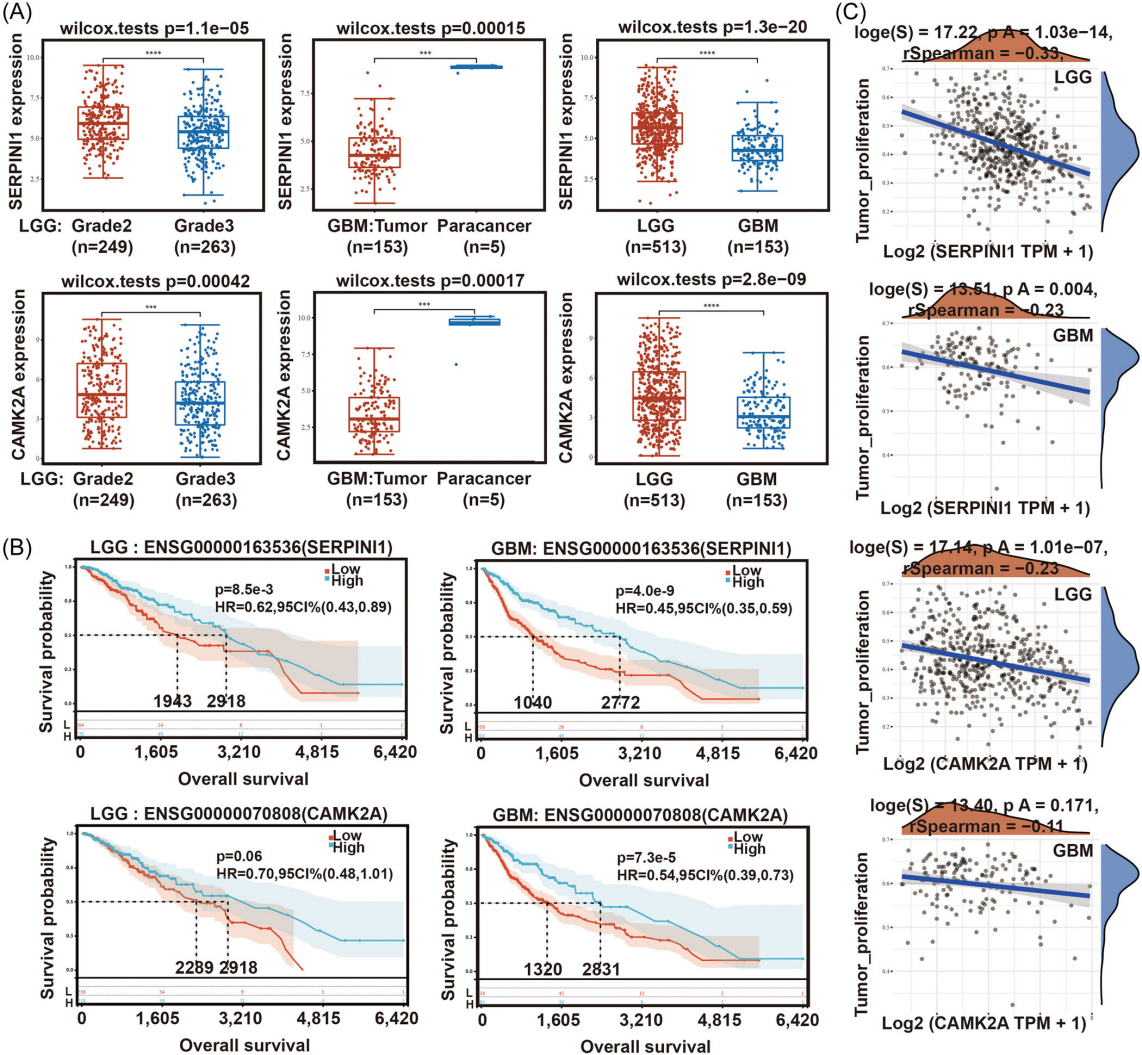
SERPINI1 and CAMK2A were negatively correlated with cell proliferation in glioma. (A) The expression differences of SERPINI1 and CAMK2A in TCGA‐LGG and ‐GBM. (B) Kaplan−Meier survival curves indicate the relationship between SERPINI1 and CAMK2A and the prognosis of patients with glioma. (C) Correlation analysis between SERPINI1 and CAMK2A expression and pathways in LGG and GBM, respectively. GBM, glioblastoma; LGG, lower‐grade glioma; TCGA, the cancer genome atlas.

To verify the role of SERPINI1 and CAMK2A, we first evaluated the expression of SERPINI1 and CAMK2A in TCGA‐LGG (Grade2 & Grade3), GBM and para‐cancer. The results revealed that high levels of both SERPINI1 and CAMK2A were low in samples with more malignancy, such as LGG: Grade3 and GBM (Figure 4A). Kaplan−Meier OS analysis indicated that SERPINI1 and CAMK2A were protective factors in patients with LGG and GBM (Figure 4B). In addition, Spearman's correlation was used to assess the relationship between SERPINI1/CAMK2A and pathway scores. The results indicated that SERPINI1 and CAMK2A were negatively correlated with tumor proliferation in LGG and GBM (Figure 4C).

### 3.5 SERPINI1 was selected as a key gene affecting the prognosis of glioma patients

**Figure 5:**
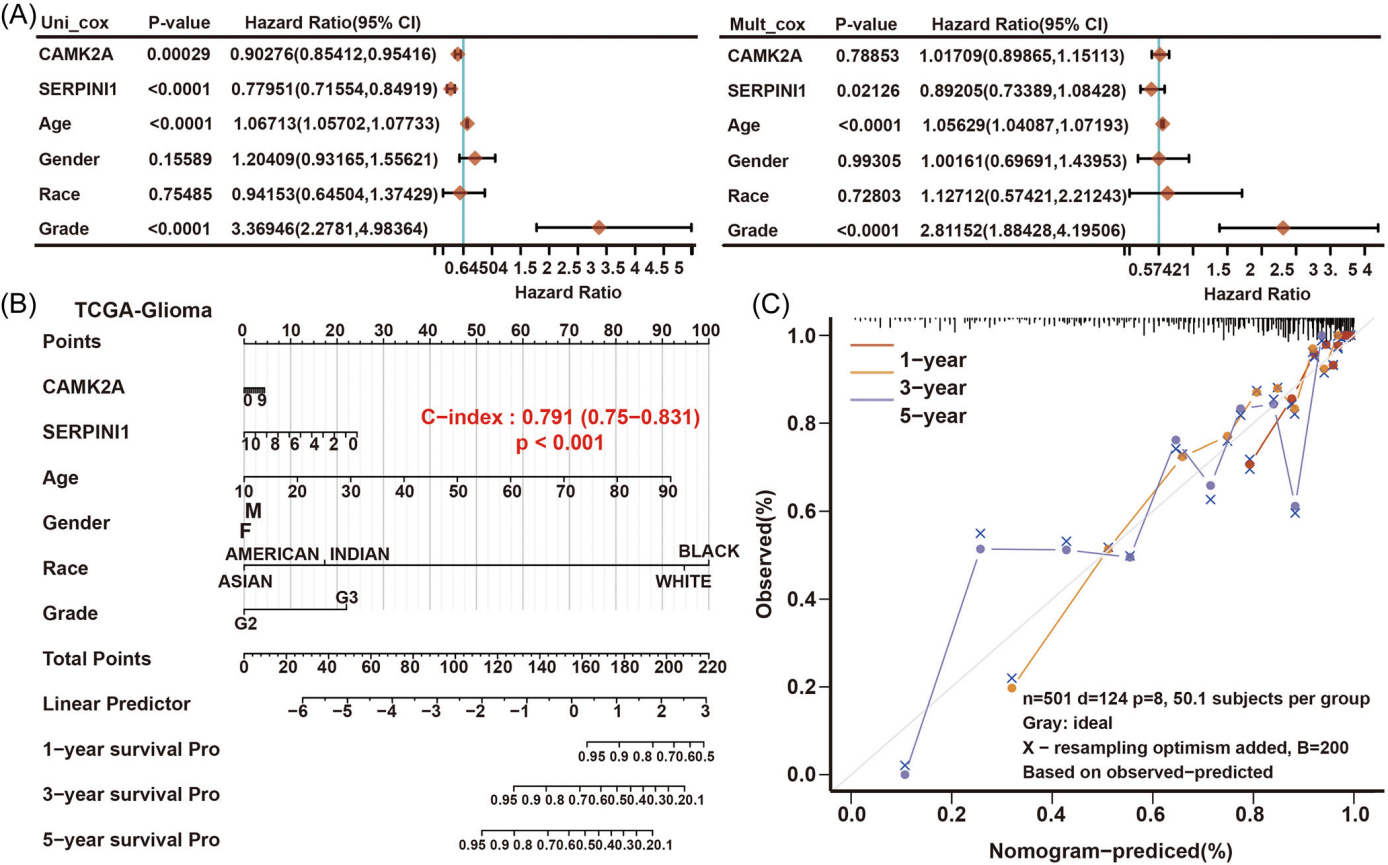
Construction of a SERPINI1‐CAMK2A‐based prognostic prediction model. (A) Univariate and multivariate Cox regression analyses of SERPINI1 and CAMK2A level with age, gender, race, and tumor stage in TCGA‐LGG and GBM cohorts. (B) Nomogram by multivariate Cox regression analysis for predicting the proportion of patients with OS. (C) These plots depict the calibration of model in terms of agreement between predicted and observed OS. GBM, glioblastoma; LGG, lower‐grade glioma; TCGA, the cancer genome atlas.

In univariate Cox regression analysis, SERPINI1 and CAMK2A expression were statistically significant. Furthermore, SERPINI1 expression was deemed an independent prognostic biomarker in the multivariate Cox proportional hazards regression model using TCGA data (for OS, HR = 0.89, 95% CI = 0.73−1.08, *p* = 0.02126) (Figure 5A).

Three independent predictors of mortality from the aforementioned analyses (SERPINI1 expression, age, and tumor grade) were combined to create a prognostic nomogram that was then tested and validated using TCGA data to better predict the prognosis of glioma patients in the clinic (Figure 5B). Subsequently, to estimate the 1‐, 3‐, and 5‐year OS rates for specific individuals, a score based on the nomogram created in the current investigation was derived. The calibration plot demonstrated that in accordance with the ideal model, the nomogram was successful in predicting patient OS (Figure 5C).

### 3.6 SERPINI1 expression could predict the clinical benefit of ICB in glioma

**Figure 6:**
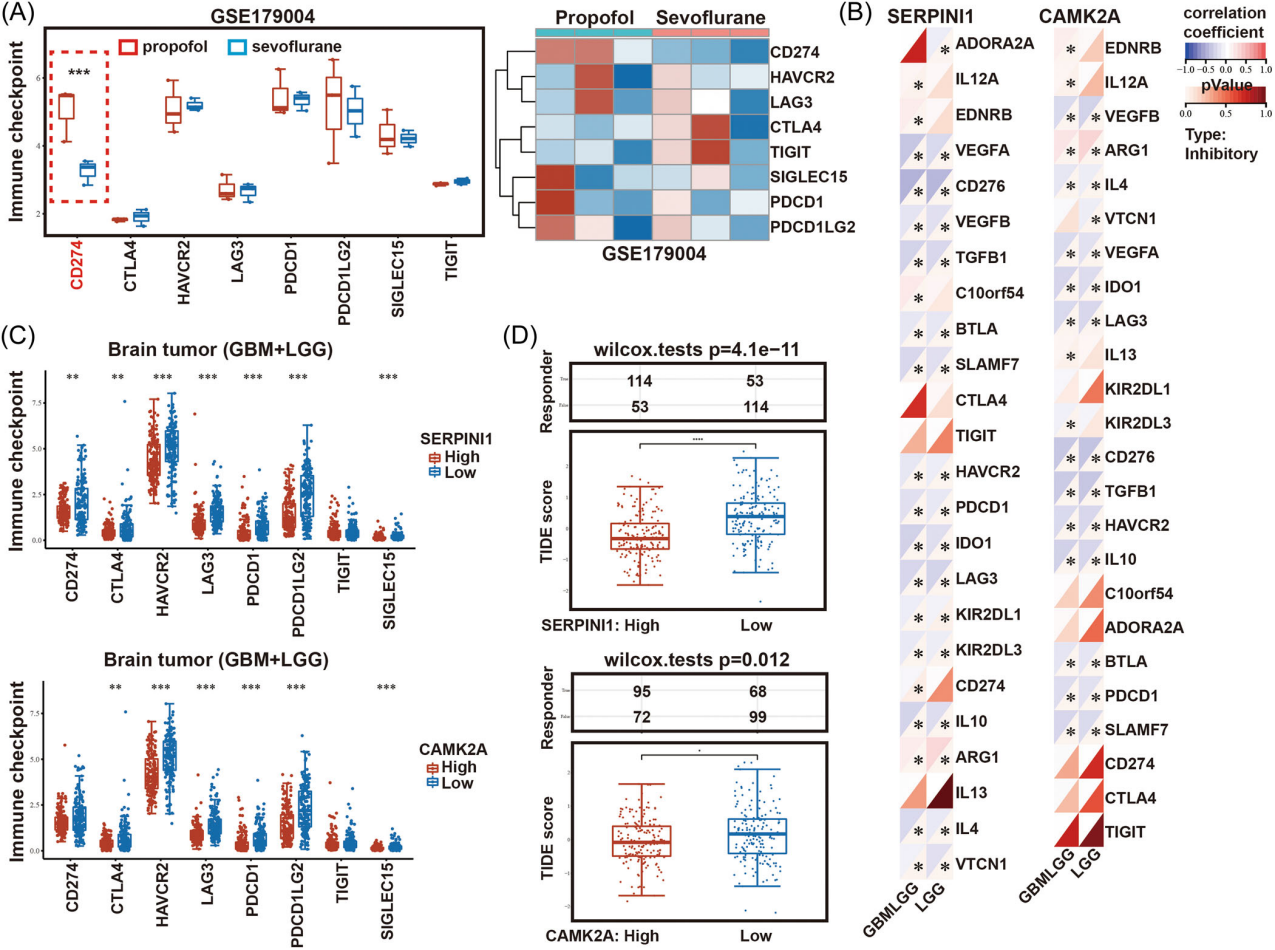
SERPINI1 expression could predict the glioma clinical benefit of ICB. (A) Compared with sevoflurane, CD274 was increased after anesthesia with propofol. (B) The negative correlation between SERPINI1 & CAMK2A expression and most immune checkpoints in TCGA‐GBM and ‐LGG cohorts. (C) The expression distribution of immune checkpoint genes in two groups (SERPINI1 & CAMK2A‐high and ‐low). (D) The distribution of immune response scores in different groups in the prediction results. Potential ICB response was predicted with TIDE algorithm. GBM, glioblastoma; LGG, lower‐grade glioma; TCGA, the cancer genome atlas.

According to the previous findings, to further explore the influence of propofol and sevoflurane on glioma immune checkpoints. We analyzed the differential effects of propofol and sevoflurane on the expression of major immune checkpoints using the GSE179004 data set. Our analysis revealed that, compared with sevoflurane, propofol significantly upregulated CD274 (PDL1), an important immune checkpoint molecule (Figure 6A). This finding suggests that propofol is more likely to suppress immune cell function and cause immune escape in gliomas.

To further explore whether SERPINI1 and CAMK2A were also associated with the expression of immune checkpoints, as shown in Figure 6B, SERPINI1 and CAMK2A expression was found to be negatively correlated with most of the immune checkpoint genes. Furthermore, we analyzed the changes in SERPINI1, CAMK2A, and immune checkpoint genes at different levels and found that high levels of SERPINI1 and CAMK2A were negatively correlated with CD274, SIGLEC15, HAVCR2, PDCD1, CTLA4, LAG3, and PDCD1LG2 expression (Figure 6C).

We further elucidated the effects of SERPINI1 and CAMK2A in the context of immunotherapy (ICBs). In our previous study, we extended our analysis to associations between SERPINI1 and CAMK2A and several immune checkpoints. Next, we investigated whether SERPINI1 and CAMK2A could predict patient responses to ICB therapy. As predicted, glioma patients with lower SERPINI1/CAMK2A expression were less likely to benefit from immune checkpoint therapy than patients with higher SERPINI1/CAMK2A expression (Figure 6D).

## 4 DISCUSSION

The standardized incidence of glioma in the Chinese population is approximately 4.11 cases per 100,000 people, of which approximately 50% are WHOIV grade GBM, which is the most common malignant brain tumor. Patients with high‐grade glioma (WHO grade III to IV) have a poor prognosis, with a median survival of 12−16 months after diagnosis.^22^ Currently, the standard treatment for newly diagnosed GBM patients consists of excision and a 6‐week cycle of external radiation therapy, combined with temozolomide (TMZ), followed by continued TMZ therapy.^23^

Compared to excision and radiotherapy alone, combined surgical excision, radiotherapy, and chemotherapy had a statistically significant survival advantage and little additional toxicity. However, the average life span of patients receiving radiation + TMZ was only 2.5 months longer than that of patients receiving radiation alone. Recurrence of LGG and other tumors is inevitable despite years of intensive study using a variety of methods in this field. Different approaches to treat this tumor are being investigated, among which immunotherapy is one of the most promising, including immunotherapy with polypeptide vaccines, oncolytic virus therapy, and immune checkpoint inhibitors.^24^, ^25^, ^26^

The transmembrane glycoprotein PD‐L1 (CD274) belongs to the B7 family of costimulatory molecules and exhibits potent immunomodulatory characteristics. Under typical physiological conditions, PD‐L1 is primarily found in myeloid cells, such as macrophages, and attaches to its receptor, programmed cell death protein‐1 (PD‐1), which is primarily expressed on activated T cells. While amplifying immunosuppressive Treg cells, PD‐1 activation prevents the growth and lysis of effector T cells.^27^ PD‐L1/PD‐1 signaling has a protective effect on T cell activation and autoimmunity in inflammatory states. However, GBM also expresses PD‐L1 and is associated with a higher tumor grade and poorer prognosis. Therefore, the PD‐1/PD‐L1 axis is an important target in glioma immunotherapy. In preclinical models, blocking PD‐L1 on glioma cells with mAb combined with radiotherapy significantly prolonged survival.^28^

Previous research has demonstrated that, after surgery, individuals with glioma frequently experience distant metastasis and tumor recurrence, which can be fatal. However, monitoring the postoperative immune system is crucial for reducing tumor recurrence and spread. Postoperative immune function is affected by the body's stress reactions, and the functional state of this system has a significant impact on the metastasis of tumor cells during surgery.^29^ An earlier investigation revealed that anesthetics affect the immune system of the body directly or indirectly by controlling immunological activity.^30^ Therefore, studying how various anesthetics affect immune cells may be helpful for therapeutic anesthesia planning. Although many studies have shown that narcotic drugs have no significant effect on overall survival and recurrence‐free survival of cancer patients, both anesthetic and analgesic drugs have varying degrees of influence on cancer cell proliferation and migration.

Sevoflurane is a widely used inhalation anesthetic in clinical practice,^31^ and has unique biological characteristics, such as fast recovery induction, controllable anesthesia depth, and organ protection. However, sevoflurane has certain effects on the proliferation and metastasis of cancer cells such as lung cancer and glioma.^32^, ^33^ LIANG et al. found that sevoflurane can downregulate HIF‐1α through p38/MAPK signaling pathway and inhibit hypoxia‐induced lung cancer cell growth and metastasis.^32^ Sevoflurane has also been observed to inhibit platelet‐induced lung cancer cell invasion via reducing platelet activity. In addition, it has been reported that sevoflurane inhibits the migration and invasion of glioma cells and enhances their sensitivity to cisplatin chemotherapy by upregulating the expression of MMP‐2 in miR‐34a‐5p.^33^ In short, both in vitro and in vivo studies and clinical studies have shown that sevoflurane can affect the biological behavior of cancer cells and the expression of some cancer factors, but sevoflurane has an impact on the overall survival or relapse‐free survival of cancer patients.

The most widely used intravenous anesthetic in clinical practice is propofol; however, its effects on the proliferation and invasion of various cancer cell types are inconsistent, and the molecular mechanism by which propofol influences the biological behavior of cancer cells remains unclear. Zhang et al. found that propofol recruited macrophages to liver cancer cells and upregulated miR‐142‐3p function by secreting microvesicles, ultimately inhibiting the metastasis of cancer cells.^34^ Clinical studies have found that propofol has no significant effect on cancer prognosis. There were no significant differences in mortality or local recurrence rates at 5 years of follow‐up after desflurane or propofol anesthesia during breast cancer surgery.^35^ In conclusion, only a few studies have demonstrated that propofol can encourage the growth of specific cancer cells, and clinical evidence suggests that propofol has little impact on cancer patients’ prognoses. However, the majority of cytological and animal studies have shown that propofol regulates different signaling cascades and noncoding RNA to prevent the proliferation, migration, and invasion of cancer cells as well as to promote cell apoptosis.^34^ Therefore, more research on the regulatory effects of propofol in various cancers and its important molecular mechanisms are still required to provide references for wise propofol selection and to conduct clinical studies to verify its effects.

According to the data we analyzed, compared with propofol, sevoflurane anesthesia is more beneficial to the recovery and prognosis of patients during glioma surgery, but the profiling alterations of metabolic, molecular, and immune modulations found in this study indicated the partial properties of sevoflurane and propofol, respectively. More in vivo and in vitro studies are needed to confirm our conclusions. More importantly, further research in clinical settings is necessary to determine the translational value of these results.

## 5 CONCLUSIONS

In summary, data from GBM samples in open databases were analyzed using bioinformatics methods, and it was discovered that propofol and sevoflurane have an impact on the gene expression of gliomas, including some immune molecules. Our current research revealed that compared with sevoflurane, propofol inhibited several metabolic pathways, such as fatty acid metabolism, the TCA cycle, and oxidative phosphorylation. However, at the same time, propofol also upregulates the immune checkpoint molecule PD‐L1 and promotes the immune escape of glioma during surgery. In contrast, we found that SERPINI1 could act as an independent prognostic factor for gliomas and was suppressed by propofol.

## AUTHOR CONTRIBUTIONS

**Shenghua Cen**: Software; validation; writing—original draft. **Guocai Yang**: Conceptualization; resources; writing—original draft. **Hongyan Bao**: Formal analysis. **Ze Yu**: Formal analysis; funding acquisition; investigation; methodology. **Lei Liang**: Investigation; resources; software; writing—review and editing.

## CONFLICT OF INTEREST STATEMENT

The authors declare no conflict of interest.

## TRANSPARENCY STATEMENT

The lead author Ze Yu, Lei Liang affirms that this manuscript is an honest, accurate, and transparent account of the study being reported; that no important aspects of the study have been omitted; and that any discrepancies from the study as planned (and, if relevant, registered) have been explained.

## Supporting information

Supporting information.

## Data Availability

The original data are available upon reasonable request to the corresponding author.
