# Supplementary material for: Impact of propofol versus sevoflurane anesthesia on molecular subtypes and immune checkpoints of glioma during surgery
Source: Health Sci Rep. 2023 Sep 11;6(9):e1366. doi: 10.1002/hsr2.1366 (PMC10495545; doi:10.1002/hsr2.1366)
Supplement: Supplementary file 1 — Supporting information. [file HSR2-6-e1366-s001.docx]

**Supplementary Methods**

*Bioinformatics analyses*

- 1. The clinical information and sample size for TCGA-GBM and -LGG dataset were came from **TCGA**. (https://portal.gdc.com)
  2. RNA-sequencing expression (level 3) profiles and corresponding clinical information for GBM and LGG were downloaded from the TCGA. R software GSVA package was used to analyze, choosing parameter as method='ssgsea'. The correlation between genes and pathway scores was analyzed by Spearman correlation (**tumor proliferation**). All the analysis methods and R packages were implemented by R version 4.0.3.
  3. **Functional Exploration of DEGs (GSE179004)**: All microarray data was downloaded from the GEO database (GSE179004). Using the limma package in the R software to study differential expressed mRNA. The GO and KEGG Enrichment Analysis are widely-used tool for annotating genes with functions.
  4. immune checkpoint analysis:

RNA-sequencing expression profiles and corresponding clinical information for glioma were downloaded from the TCGA dataset. SIGLEC15, TIGIT, CD274, HAVCR2, PDCD1, CTLA4, LAG3 and PDCD1LG2 were selected to be immune-checkpoint–relevant transcripts and expression values of these eight genes were extracted. All the above analysis methods and R package were implemented by R foundation for statistical computing (2020) version 4.0.3. Using the ggplot2 R package and pheatmap R package.

- 1. ICB response:

The expression of ALDOB across TCGA cancers (with tumor and normal samples)

RNA-sequencing expression profiles and corresponding clinical information for glioma were downloaded from TCGA. Potential ICB response was predicted with TIDE algorithm.

**Abbreviations**: GEO, Gene Expression Omnibus; DEG, Differentially expressed gene; TCGA, The Cancer Genome Atlas; GBM, Glioblastoma multiforme; LGG, brain Lower grade glioma; WGCNA, Weighted correlation network analysis; GO, Gene Ontology; KEGG, Kyoto Encyclopedia of Genes and Genomes; TIDE, Tumor Immune Dysfunction and Exclusion; ICB, immune checkpoint blockade;

**Supplementary Figure and Figure legend**

**
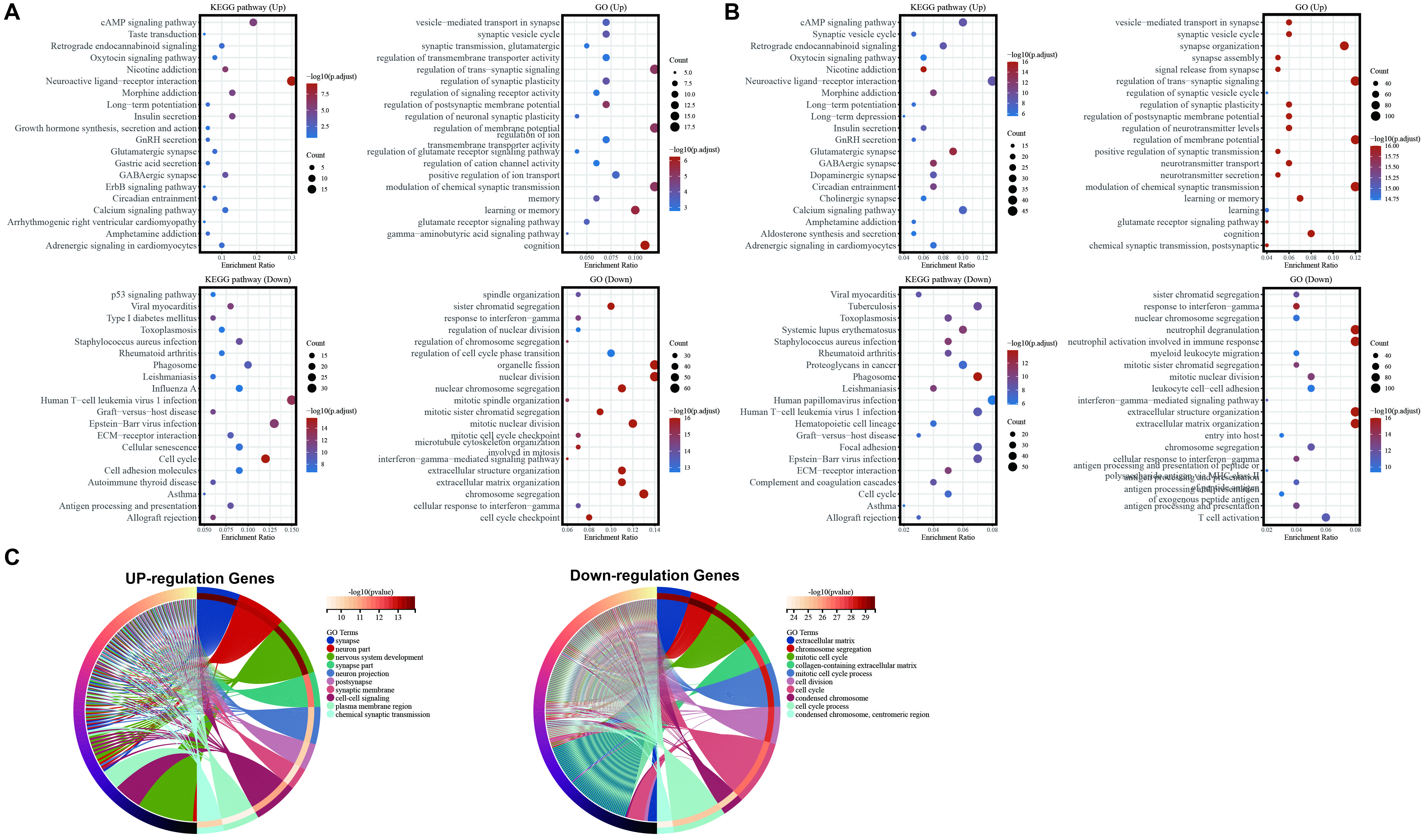
**

**Supplementary Figure 1** KEGG pathway enrichment and Gene ontology analysis of the DEGs

**(A, B)** KEGG pathway enrichment and Gene ontology analysis of DEGs based on LGG Grade2 vs. Grade3; and LGG vs. GBM. (**C**) Significantly enriched the GO analysis of the DEGs obtained and visualized through cluster-Profiler.
